# Transmembrane signaling and cytoplasmic signal conversion by dimeric transmembrane helix 2 and a linker domain of the DcuS sensor kinase

**DOI:** 10.1074/jbc.RA120.015999

**Published:** 2020-12-10

**Authors:** Marius Stopp, Philipp Aloysius Steinmetz, Christopher Schubert, Christian Griesinger, Dirk Schneider, Gottfried Unden

**Affiliations:** 1 Microbiology and Wine Research, Institute for Molecular Physiology, Johannes Gutenberg University Mainz, Mainz, Germany; 2 Department of NMR-based Structural Biology, Max Planck Institute for Biophysical Chemistry, Gottingen, Germany; 3 Department of Chemistry, Biochemistry, Johannes Gutenberg University Mainz, Mainz, Germany

**Keywords:** sensor kinase, DcuS, fumarate, transmembrane signaling, (Gly)xxx(Gly) motif, piston-type, linker, oxidative Cys cross-linking, CL, cross-linking, C4DC, C_4_-dicarboxylate, GpA, glycophorin A, MBP, maltose-binding protein, TM, transmembrane

## Abstract

Transmembrane (TM) signaling is a key process of membrane-bound sensor kinases. The C_4_-dicarboxylate (fumarate) responsive sensor kinase DcuS of *Escherichia coli* is anchored by TM helices TM1 and TM2 in the membrane. Signal transmission across the membrane relies on the piston-type movement of the periplasmic part of TM2. To define the role of TM2 in TM signaling, we use oxidative Cys cross-linking to demonstrate that TM2 extends over the full distance of the membrane and forms a stable TM homodimer in both the inactive and fumarate-activated state of DcuS. An S_186_xxxGxxxG_194_ motif is required for the stability and function of the TM2 homodimer. The TM2 helix further extends on the periplasmic side into the α6-helix of the sensory PASP domain and on the cytoplasmic side into the α1-helix of PAS_C_. PAS_C_ has to transmit the signal to the C-terminal kinase domain. A helical linker on the cytoplasmic side connecting TM2 with PAS_C_ contains an LxxxLxxxL sequence. The dimeric state of the linker was relieved during fumarate activation of DcuS, indicating structural rearrangements in the linker. Thus, DcuS contains a long α-helical structure reaching from the sensory PAS_P_ (α6) domain across the membrane to α1(PAS_C_). Taken together, the results suggest piston-type TM signaling by the TM2 homodimer from PASP across the full TM region, whereas the fumarate-destabilized linker dimer converts the signal on the cytoplasmic side for PAS_C_ and kinase regulation.

Membrane-anchored bacterial histidine kinases typically perceive ambient stimuli *via* their extra-cytoplasmic sensor domains (1, 2). Intracellularly, the signal is forwarded *via* cytoplasmic PAS, HAMP, or GAF domains to the kinase domain. The structure and function of several extra-cytoplasmic sensor domains have been studied previously, including the sensor domains of the CitA, DcuS, NarX/Q, and PhoQ sensor kinases (3, 4, 5, 6, 7). The structure and function of the cytoplasmic signal-transducing and kinase domains have also been studied (7, 8, 9, 10, 11, 12). The exact mechanism of transmembrane (TM) signaling by sensor histidine kinases is still enigmatic, but evidence for diverse mechanisms, including piston-type movement of TM helices, scissor-like kinking with structural rearrangement, rotation of the TM helices, or a combination of these, has been obtained (6, 7, 13, 14, 15, 16, 17, 18, 19, 20, 21).

The fumarate, or C_4_-dicarboxylate (C4DC), sensor kinase DcuS of the DcuS–DcuR two-component system consists of a PAS_P_ (Per-ARNT-SIM) sensor domain, a TM domain composed of the antiparallel transmembrane helices TM1 and TM2, and a cytoplasmic PAS_C_ and the kinase domain (22, 23). The sensory PAS_P_ domain is framed by TM1 and TM2 and adopts periplasmic location. For C4DC responsiveness, DcuS requires C4DC transporter DctA or DcuB under aerobic and anaerobic conditions, respectively (24, 25, 26). DcuS forms a homodimer in bacterial membranes (27), and the two α-helices TM1 and TM2 of the DcuS monomer are supposed to facilitate DcuS homodimerization (18). DcuS TM signaling involves TM2, which is linked on the periplasmic side to the PAS_P_ domain and on the cytoplasmic side to the PAS_C_ domain. Binding of a C4DC ligand to the sensor domain (28, 29, 30) results in compaction of the ligand binding site in PAS_P_ and uplifting of the C-terminal α6-helix of PAS_p_. The uplift and structurally related arrangements upon citrate binding have been shown directly for homologous CitA in a membrane context (6, 21). This uplift pulls the periplasmic part of TM2 of DcuS in the direction of the periplasm in a piston-like movement (18). TM signaling by sensor kinases is, however, complex and can change in different segments of the TM region or combine different modes of conformational changes (31). In contrast, TM1 of DcuS exhibits no piston movement and revealed no contribution to TM signaling (18).

To test for piston-type movement or indications for additional modes of TM signaling upon fumarate activation of DcuS, we studied the α-helical arrangement and dimerization of TM2 for the full-length and for the adjacent cytoplasmic region by Cys scanning and oxidative cross-linking (CL). This should define the extension of the α-helix from the periplasmic side across the membrane and its connection to the cytoplasmic PAS_C_ domain, with an emphasis on structural properties that could be relevant for the signal transfer across the membrane. Cysteine scanning and oxidative CL were applied as major methods to study TM2 interactions, as well as structural alterations due to receptor activation by fumarate. All studies were performed in bacteria containing DctA, which is a functional determinant of DcuS function (25).

## Results

### TM2 of DcuS homodimerizes at specific contact sites regardless of activity state

**Figure 1:**
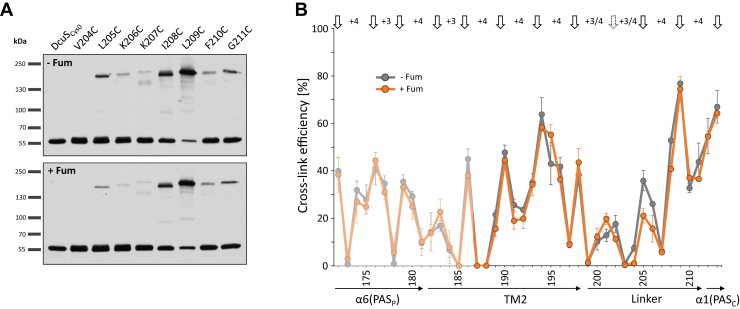
**Oxidative *in vivo* Cys disulfide cross-linking in the α6(PAS**_**P**_**)-TM2-linker-α1(PAS**_**C**_**) region of DcuS.***A*, western blots for CL in *E. coli* C43 cells containing DcuS single-Cys variants at positions Val204–Gly211 and the Cys-less control (DcuS_Cys0_). Cross-linking was performed by adding Cu^2+^ phenanthroline to the reaction mixture in the absence of fumarate (–Fum) and in the presence of 50 mM fumarate (+Fum). The complete set of western blots for the Cys variants from positions Val187 to Glu213 are shown in Figs. S7 and S8. *B*, the ratio of the cross-linking product to total DcuS was calculated by scanning the western blots and quantitatively evaluating the signal in ImageJ software by measuring the band intensities. The CL efficiency for each individual Cys residue was determined in three independent experiments and the arithmetic mean plotted. CL depended completely on the addition of Cu^2+^ phenanthroline. The maxima of CL efficiencies are highlighted by *arrows* and their spacing is indicated. Values for Val172–Ser186 (presented in *light lines*) from Monzel and Unden (18) are included for completeness.

**Figure 2:**
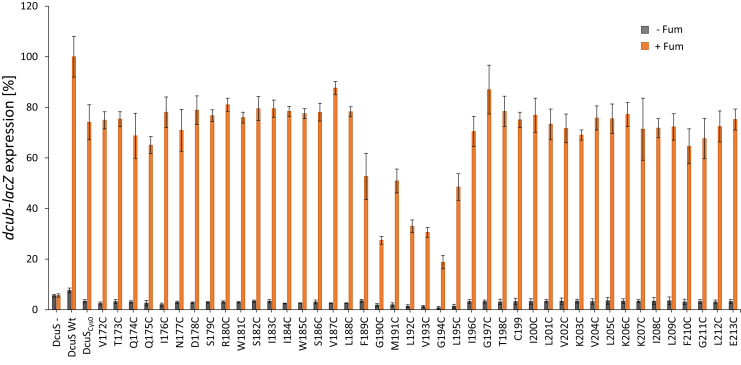
**Effect of DcuS cysteine substitutions in the TM2-PASC linker region on *dcuB-lacZ* expression.** Expression of *dcuB* and effect of the substitutions were tested in the *dcuS* negative strain IMW260 (DcuS–) complemented with plasmid (pMW336)-encoded Cys-less DcuS (DcuS_Cys0_) and derivatives of DcuS with single Cys substitutions. Growth was performed under anaerobic conditions in eM9 medium with glycerol plus 20 mM DMSO with or without 20 mM disodium fumarate. All activities were normalized to the wild-type control of DcuS in the fumarate-activated state. Variants 172–188 and 196–213 have been tested earlier (18) without presenting the data.

The role of TM2 and the adjacent cytoplasmic region in DcuS homodimerization was analyzed in aerobically grown *Escherichia coli* cells, i.e., in the native context with DctA, by Cys CL using membrane-permeant copper(II)-(1,10-phenanthroline)_3_, or “Cu^2+^ phenanthroline,” as an oxidant (18, 32, 33). In our previous study, we used CL to study interactions between α6 of PAS_P_ and the initial part of TM2 up to residue Ser186 (18). Here, we studied the involvement of every single residue of the complete TM2 helix in DcuS dimerization up to residue Glu213 (Fig. 1), which is located on the cytoplasmic side in the N terminus of the α1-helix in PAS_C_ (9, 34). Each amino acid was individually replaced genetically by a Cys residue in the plasmid-encoded Cys-less variant DcuS_Cys0_. In DcuS_Cys0_, the natural Cys199 and Cys471 are replaced by Ser, which only moderately decreases fumarate stimulation of DcuS (18, 27). The Cys variants retained all 87%–118% of DcuS_Cys0_ activity in *dcuB-lacZ* expression and induced *dcuB-lacZ* in a fumarate-dependent manner as the wild-type (Fig. 2). However, there was a striking drop in *dcuB-lacZ* expression in a coherent stretch of seven amino acids (F189–L195).

Cys-mediated CL of the DcuS variants was visualized by nonreducing SDS-PAGE and Western blotting with DcuS specific antisera (18), which allowed us to separate DcuS monomers (calculated mass 62 kDa; apparent mass ∼55 kDa for His_6_-DcuS) from cross-linked DcuS homodimers (calculated mass 124 kDa; apparent mass ∼170 kDa; Fig. 1*A*). Reaction of the antiserum with DcuS and the cross-link product was verified by comparing the reaction with purified DcuS (Fig. S1) (35).

The CL efficiency was calculated from the levels of cross-linked *versus* free DcuS (Fig. 1*A*) and ranged from 0 to 74%. We ensured that the band intensity was in the linear range of detection to allow a comparison of band intensities (18). The CL efficiencies were high at several positions within TM2 (approx. Ser182 to Leu201), the periplasmic α6 of PAS_P_, and the cytoplasmic α1-PAS_C_ helices (see Fig. 1*B*). Lower CL efficiency was observed at the membrane/water interfaces on the periplasmic (residues Trp181–Trp185) and cytoplasmic (Cys199–Lys207) sites. For most regions, a +3/+4 periodicity of high CL efficiency was observed (Fig. 1*B*), which is characteristic of the interaction between two α-helices. Notably, the CL efficiency and the +3/+4 periodicity were retained in the region from F189 to L195, demonstrating that the region has a supposed α-helicity, which is retained in the Cys replacement mutations.

Remarkably, the CL pattern and yield were essentially identical for fumarate activated *versus* nonactivated DcuS, with a maximal 12% difference in the CL efficiency. The clearest differences were observed in the Cys199–Lys207 region, corresponding to the TM2-PAS_C_ interdomain or linker region. In this region, a generally low CL efficiency was observed with partial nonhelical spacing of the CL maxima, and relatively large fumarate-induced differences, especially for residues Leu201, Val202, Val204, Leu205, and Lys206.

The CL results suggest that the region from α6 in PAS_P_ to α1 in PAS_C_ forms a continuous α-helix, including the complete TM2 region.

### A GxxxG motif in TM2: its role for DcuS activity and DcuS homodimerization

The sensitivity of DcuS activity to Cys replacement mutation in the F189–L195 region coincides with the presence of a GxxxG motif in this region. GxxxG motifs frequently mediate the interactions of TM α-helices *via* “ridge into groove” tight packing of two interacting TM helices (36, 37, 38). The Gly residues can be replaced by other amino acid residues with small side chains, such as Ala, Ser, or Thr, resulting in a (small)xxx(small) (39) or glycine-zipper-like motif, (small)xxx(small)xxx(small) (40). The F189–L195 region in TM2 is part of such a glycine-zipper-type motif S_186_xxxGxxxG_194_ (Fig. S2) (41, 42).

**Figure 3:**
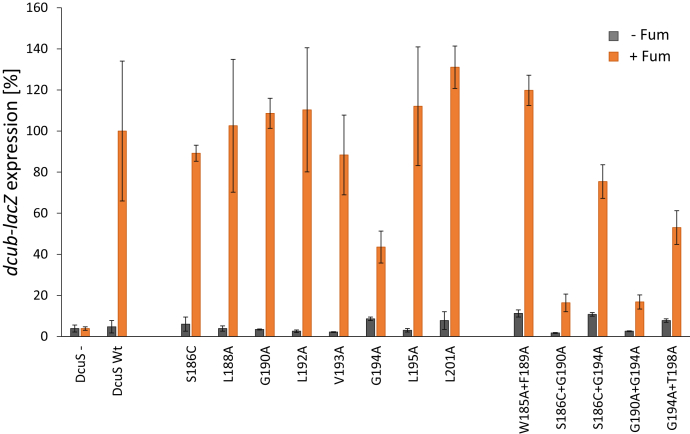
**Effect of single and double mutations in the DcuS TM2 region on *dcuB-lacZ* expression.** The DcuS variants were tested in *dcuS*-negative *E. coli* IMW260 (DcuS–) complemented with plasmid (pMW181)-encoded wild-type DcuS (DcuS Wt) or DcuS variants (Table S1) (45, 46, 47, 48). The effect is shown for single (center) and double (right) mutants. Growth was performed under anaerobic conditions in eM9 medium with glycerol plus DMSO with or without 20 mM disodium fumarate. Activities were normalized to the wild-type control of DcuS in the fumarate-activated state. Three biological replicates were tested and the error bars indicate SD.

A role of the motif for DcuS function and activity was tested by substituting the motif residues and adjacent residues with Ala or Cys (for Ser), which avoids gross structural rearrangements. Replacement of Gly with Ala in GxxxG motifs disrupts helix–helix interactions, as the Gly residues are important for TM helix dimerization (38, 43, 44). The impact of the mutations on DcuS function was assayed using the variants in a DcuS-less background by monitoring DcuS-dependent *dcuB-lacZ* reporter gene expression (Fig. 3). Individual replacements in the signature residues Ser186 and Gly190 of the motif did not significantly affect *dcuB-lacZ* expression, whereas the activity decreased to 44% of wild-type after replacement of Gly194 by Ala. Mutation of other residues in that region had no effect as well. However, mutation of the core pair of the motif (Gly190 residue together with Gly194) dramatically reduced the activity to ∼16% of wild-type. The effect is specific since mutation of neighboring residues (W185A together with F189A) had no effect. Remarkably, mutation of Ser186 together with Gly190 showed the same reduction in activity as for the Gly190 plus Gly194 pair. Taken together, the data suggest that the S_186_xxxGxxxG_194_ motif in TM2 (particularly Gly190 or the Gly190 + G194 pair) is crucial for DcuS function. Notably, the level of DcuS protein in membranes was similar for wild-type and all variants after expression (Fig. S3*A*), confirming that the observed differences were caused by the mutations rather than by altered DcuS levels.

As Thr residues can also be a part of (small)xxx(small) motifs (38), we analyzed the involvement of Thr198. However, when both the Thr198 and the Gly194 residues were replaced by Ala residues (Fig. 3), *dcuB-lacZ* expression was not decreased compared with DcuS(G194A) alone, indicating that Thr198 is not important for DcuS activity.

### Role of the S_186_xxxGxxxG_194_ motif in homodimerization of TM2

**Figure 4:**
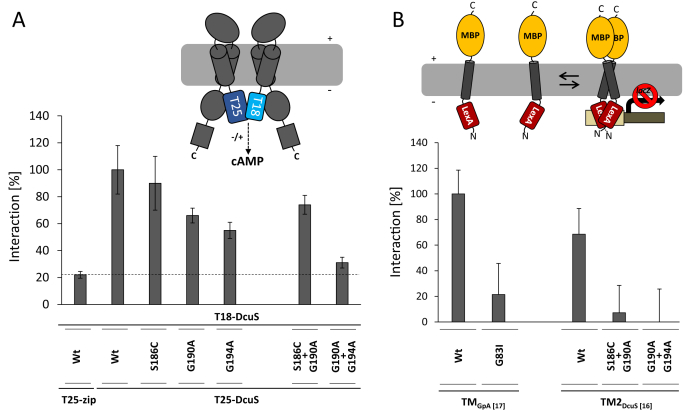
**Involvement of the S**_**186**_**xxxGxxxG**_**194**_**motif in TM2 homodimerization of DcuS and isolated TM2.***A*, in the BACTH system, *E. coli* BTH101 cells were cotransformed by plasmid pairs encoding T18-DcuS (pMW429), T25-DcuS (pMW426), and its derivatives encoding the single and double variants of the S_186_xxxGxxxG_194_ motif of DcuS. The cells were grown aerobically to an OD_578_ of 0.5–0.7 and the β-galactosidase activity assayed. All values were normalized to the value for the interaction between the T18-DcuS and T25-DcuS Wt pair. The activity of the noninteracting T25-Zip and T18-DcuS pair was defined as the background. *B*, *E. coli* SU101 was transformed with GALLEX constructs encoding TM2 of DcuS with a length of 16 aa (TM2_DcuS [16]_; sequence in Fig. S4). The bacteria were cultivated aerobically in LB to an OD_578_ of 0.5 and β-galactosidase activities determined. The dimerization of TM2_DcuS[16]_ (G190A + 194A), which exhibited the weakest inhibition of β-galactosidase expression, was set to 0%, and all other values were normalized accordingly. The constructs TM_GpA [17]_ (Wt) and TM_GpA [17] (G83I)_ served as controls for interaction in the GALLEX system (51). Three biological replicates were tested in (*A*) and (*B*) and the error bars indicate SD.

The role of the S_186_xxxGxxxG_194_ motif in DcuS homodimerization was tested using bacterial two-hybrid (BACTH) systems. For the full-length DcuS protein, we applied the BACTH system that relies on the restoration of adenylate cyclase activity and cAMP production by fusing the individual domains (T18 or T25) of *Bordetella pertussis* adenylate cyclase to interacting proteins. Interaction restores the adenylate cyclase activity, which can be monitored as β-galactosidase expression (49, 50). The T18 and T25 domains were genetically fused to the DcuS N terminus. When T18-DcuS was coexpressed with T25-DcuS, high β-galactosidase activity was observed for wild-type DcuS (Fig. 4*A*). When the signature residues Gly190 and Gly194 of the S_186_xxxGxxxG_194_ motif were individually mutated, activity (DcuS homodimerization-dependent *lacZ* expression) decreased to ∼70% and ∼55%, of wild-type for the G190A and G194A variants, respectively, but did not significantly decrease for the S186C variant. Combining the Gly190 with the Gly194 mutation to the G190A + G194A double mutant decreased the activity to near background levels. Combination of S186C with the G190A mutation to the S186C + G190A double mutant added no further effect to the single G190A mutant. The levels of T18- and T25-fusion proteins and their membrane integration were confirmed to rule out that changes in activity were caused by low expression or lack of membrane integration (Fig. S3). Taken together, these data suggest that the central G_190_xxxG_194_ pair within the S_186_xxxGxxxG_194_ motif is important for homodimerization of DcuS.

We applied the GALLEX two-hybrid system to test TM2 homodimerization in the absence of other, potentially interfering, domains of DcuS (51, 52). In the GALLEX system, the C termini of isolated TM domains are genetically fused to the maltose-binding protein (MBP) that ensures proper membrane integration of the TM helix. At the TM2 N terminus, the DNA-binding domain of the LexA protein was fused (Fig. 4*B*). Dimerization of DcuS TM2 would result in dimerization of the DNA-binding domain, which then binds to the *sulA* promoter, resulting in repression of the downstream *lacZ* reporter gene (53) and decreased β-galactose activity (Fig. 4*B*). The strongly interacting TM region of human glycophorin A (GpA) serves as a positive and the weakly interacting variant GpA(G83I) as a negative control (51). First, a 16-amino-acid helix (Ile183–Thr198) was established as the optimal length of DcuS TM2 for GALLEX analysis, and the expression, membrane insertion, and proper membrane topology of the expressed fusion protein were confirmed (Figs. S4 and S5). Expression of the wild-type TM2-LexA strongly inhibited *lacZ* expression, meaning that the isolated TM2 of DcuS strongly homodimerized with about 70% of GpA wild-type (Fig. 4*B*). However, the observed interaction (measured as inhibition of the β-galactosidase activity) was lost in the case of DcuS TM2 double-mutant G190A + G194A but also S186C + G190A (Fig. 4*B*). The activities of the double mutants even fell below the negative control of the GpA (G83I) variant. These results confirm that TM2 of DcuS homodimerizes, which is in agreement with the BACTH and CL results, and show that the S_186_xxxGxxxG_194_.motif plays an important role in the homodimerization. Though the S186C + G190A mutation did not dramatically affect dimerization of full-length DcuS in the BACTH assay (Fig. 4*A*), homodimerization of isolated TM2 was affected nearly as dramatically as in the case of the G190A + G194A mutant (Fig. 4*B*). The retained interaction in the S186C + G190A mutation in the BACTH assay indicates that other DcuS regions further stabilize the TM2-mediated interaction in the full-length protein. Overall, the BACTH and GALLEX data demonstrate an important role of the S_186_xxxGxxxG_194_ motif, and particularly G_190_xxxG_194_, in homodimerization of isolated TM2 and full-length DcuS.

**Figure 5:**
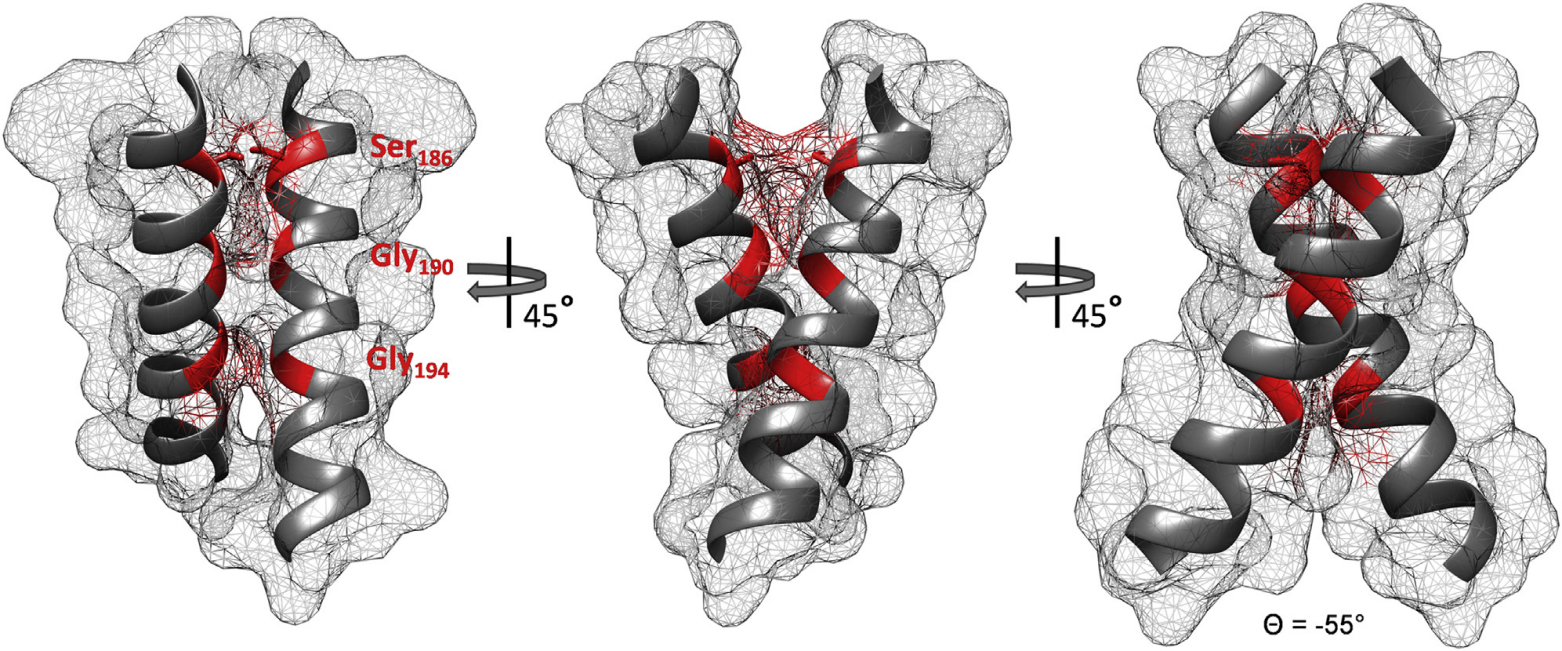
**Model of the TM2 homodimer and the location of Ser186, Gly190, and Gly194 of the S**_**186**_**xxxGxxxG**_**194**_**motif.** The structural model of a TM2 homodimer was generated using the PREDDIMER web tool (54) with the input sequence from DcuS position Ile_176_ to Phe_210_. The helices are limited to residues Ser182–Leu201. The membrane–water interfaces at the periplasmic and cytoplasmic sides are at Ser182 and Cys199, respectively. The positions of Ser186, Gly190, and Gly194, as well as the predicted contact sites of Ser186, Gly190, and Gly194 in the surface projections of the TM2 homodimer interface, are marked in *red*. The crossing angle of the predicted TM2 homodimer (Θ) is shown below the right projection.

The CL data and the interaction studies indicate a TM2/TM2ʹ homodimer that is stabilized by the S_186_xxxGxxxG_194_ motif, which forms the TM2/TM2ʹ interaction surface. Calculation of the TM2/TM2ʹ homodimer by the web-tool PREDDIMER (54) provided as one of the top-scoring structures a homodimer with an interaction surface that involves exactly the residues S_186_xxxGxxxG_194_ motif (Fig. 5). Thus, based on the experimental data and predicted structure, the TM2 helix forms a right-handed dimer with a crossing angle of –55°. Residue Thr198 that is located on the subsequent helical turn of the dimer also exhibits a high degree of Cys-CL (Fig. 1*B*), and Thr residues can be part of (small)xxx(small) motifs (38). Mutation studies did not prove direct involvement of Thr198 in TM2 homodimerization and function, and Thr198 was not prominently involved in formation of the dimer interface in the structural model (Fig. 5). Therefore, Thr198 appears to be in spatial proximity, as shown by Cys-CL, but not directly involved in helix dimerization, which is in line with the crossing angle of 55° (Fig. 5).

### The TM2-PAS_C_ linker is involved in regulation of DcuS activity

The linker connecting the C terminus of TM2 (18) with α1 of PAS_C_ (34) ranges from Cys199 to Gly211 (Fig. 1). This linker (C_199_ILVKVLKKILFG_211_) includes the LxxxLxxxL sequence. Sequences with Leu spacings of this type are found in interactions between proteins regulating diverse cellular functions (55). The linker is located at the membrane–cytoplasm interfacial region of TM2. Leu205 and Leu209 of the linker attracted attention when the piston mode of TM signaling by TM2 was identified (18). Upon the activation of DcuS by fumarate, Leu205 becomes inaccessible and Leu209 accessible to labeling by hydrophilic reagents, which was interpreted to reflect structural reorganization such as a move into or out of the hydrophobic membrane core region (18). Remarkably, three Lys residues are present in the linker, which is in agreement with the cytoplasmic location and positive inside rule (18, 56). As seen in Figure 1, the residues of the linker could be cross-linked when individual residues were replaced by Cys. When the linker residues were mutated individually to Ala, a major loss in DcuS function (in the presence of fumarate) was observed for Leu209, and to a lower extent also for the adjacent Phe210, in the *dcuB-lacZ* reporter gene assay (Fig. S6). In contrast, substitution of Lys206 by Ala resulted in a clear ON phenotypes in the *dcuB-lacZ* reporter gene assay in the absence of fumarate, which was not observed when mutating the remaining residues, apart from a weak ON phenotype of the V202A substitution. Remarkably, the Cys substitutions in the linker used for the CL assay were not conspicuous in the DcuS activity assay (Fig. 2), suggesting that Cys substitutions are more neutral than Ala substitutions in this region. Cys replacements in the region connecting the TM region of the chemotaxis protein Tsr with the signal transducing HAMP domain, or “control cables,” also showed a remarkable tolerance to Cys substitutions (57).

The strong effects observed upon mutation of Lys206 and Leu209 suggest that the linker is important for controlling DcuS activity. This assumption is further supported by the observation that the CL efficiency in the linker (Cys199–Lys207) varied to some extent in the presence and absence of fumarate (Fig. 1*B*).

### Cross-linking dynamics of TM2 and the linker

**Figure 6:**
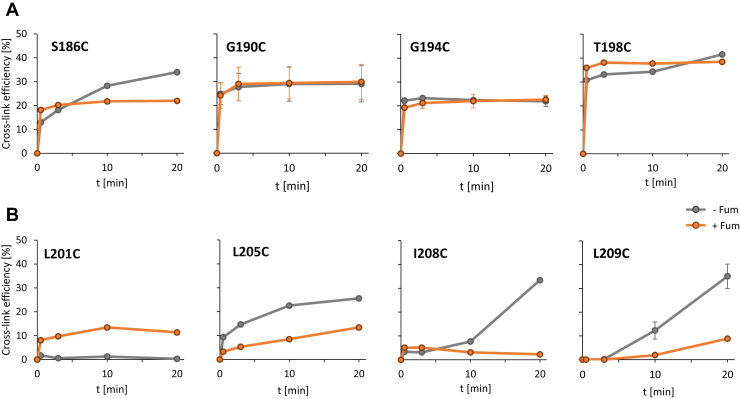
**The effect of fumarate on oxidative Cys CL of sites in TM2 (*A*) and the linker (*B*): Time-resolved CL.** The bacteria for the *in vivo* CL assay were grown in the presence (*orange*) or absence (*gray*) of fumarate. Oxidative CL conditions were performed under optimized conditions with respect to Cu^2+^ phenanthroline concentrations and temperature with and without fumarate. For key residues G190C, G194C, and L209C, the CL kinetics was performed in repeat, and error bars are given. The ratio of CL products to the total amount of DcuS was calculated after scanning the anti-DcuS western blots and evaluating the band intensities in ImageJ software. The western blots are shown in Figure S9.

Cys-CL of TM2 and the linker (Fig. 1) was performed under relatively strong oxidizing conditions (16, 32) to obtain clear information on α-helicity. The CL indicated a decrease in efficiency in the linker region compared with TM2 and some differences in the sensitivity to fumarate for the linker and the TM2 regions (Fig. 1*B*). The differences were resolved in the following experiments in more detail (Fig. 6). To this end for each residue, optimal oxidation conditions were adjusted by optimizing the concentration of Cu^2+^ phenanthroline and temperature in order to achieve a decrease in oxidative reactivity (see Experimental procedures section). This enabled monitoring CL efficiency over time and comparison for the presence or absence of fumarate. DcuS variants with high CL yield (Fig. 1*B*) were chosen for analysis to enable quantitative evaluation even under weakened oxidizing conditions. For the TM2 region, amino acids Ser186, Gly190, Gly194, and Thr198 were selected (Fig. 6*A*). For positions Gly190, Gly194, and Thr198, high CL levels were observed within short time (<1 min) after addition of the oxidant, and the kinetics were not affected by the presence of fumarate. Thus, the interacting Cys residues are similarly positioned in both the inactive and fumarate-activated state. DcuS(S186C) was also rapidly cross-linked with and without fumarate, but the CL efficiency increased slowly in the absence of fumarate.

The CL kinetics were clearly different for residues in the linker region (Fig. 6*B*). For Cys residues at Leu205, Ile208, and Leu209, the CL efficiency was very low after 0.5–3.0 min in the presence of fumarate, and only slightly increased, if at all, within 20 min. However, in the absence of fumarate, the CL efficiency was also low at the beginning but increased over time. After 20 min, the CL efficiency of the fumarate-deficient state was increased by a factor of 2–7 compared with the fumarate-activated state. The increase was particularly evident for the C-terminal part of the linker (residues 208 and 209). Therefore, the CL conditions allowed a clear differentiation of efficiency for the individual residues in response to fumarate. At position L201, where the linker emanates from TM2 close to the membrane/cytoplasm interface, the CL response was different from those seen for other positions in the linker. CL in the presence of fumarate was low but reached maximal levels very early after 1 min, similar to the neighboring TM2 region (Fig. 6*A*).

Therefore, the data suggest major differences for homodimerization of TM2 and the linker region, as residues of the linker region (Leu201, Leu205, Ile208, and Leu209), but not the TM helix, exhibited a marked fumarate-induced difference in CL efficiency. These findings suggest fumarate-induced effects on helix dimerization and restructuring of (at least) the extra-membranous part of the linker.

PAS_C_ starts at Leu212 with a long helical region (α1) and nearly parallel arrangement in the PAS_C_ dimer (9, 34). Since α1 directly connects the linker to PAS_C_, the CL efficiency and fumarate response were tested and compared with the response of the linker. When residues of the α1 region immediately following the linker were individually mutated to Cys, a +4 pattern was observed in CL in accordance with the presence of canonical α-helices. The CL pattern was retained in the inactive and fumarate-activated state of DcuS (Fig. 1*B*). Therefore, the linker located between TM2 (Ser182 to Cys199) and PAS_C_ starting at Leu212 differs from both regions by its CL efficiency and its response to fumarate. The linker has to be considered as a separate region with distinct properties.

## Discussion

In the present study, we show that DcuS contains a long continuous α-helix connecting the periplasmic PAS_P_ sensor domain with PAS_C_ on the cytoplasmic side of the membrane. The studies were performed in bacteria to ensure the availability of functional DcuS, which requires the presence of transporter DcuB or DctA (24, 25). The α-helix starting at Val172 in PAS_P_ and ranging to Glu213 in PAS_C_ comprises 9–10 helical turns. The major part (Val172–Cys199) remains homodimeric as shown by Cys scanning and CL study for the full length of the α-helix, regardless of the presence or absence of fumarate, indicating a rigid dimer from PAS_P_ across the membrane to the linker. This TM2 helix dimer is stabilized by the S_186_xxxGxxxG_194_ motif, which forms the TM2/TM2ʹ interaction surface. The role of the motif was consistently verified by mutational, functional, and interaction studies and structural modeling of the region. The data show also that the core of the motif (G_190_xxxG_194_) is extended to obtain the tandem S_186_xxxGxxxG_194_ motif. The stability of the TM2/TM2ʹ dimer suggests its parallel movement upon fumarate activation. It is not known whether both PAS_P_ domains of the dimer are fumarate-bound for activation. For full activation, high levels of fumarate (50 mM) were applied, indicating that both monomers are fumarate-bound, given the app. *K*_*M*_ values of approx. 2 mM for C4DCs (28).

Piston-type movement toward the periplasmic side upon fumarate activation has been shown for the N-terminal portion of TM2 (18). The stability and persistence of the TM2 helix dimer demonstrated here strongly suggest a piston-type shift of the entire TM2 dimer for the full TM region upon activation. The persistence of the dimer contact sites is in line with a parallel piston movement. It provides strong arguments against rotational movement of TM2 in TM signaling and against major scissor-like movements in TM2. Scissoring requires kinking and reorganization of α-helices, which is not observed here. Furthermore, Pro residues, which support kinking and bending (14, 16, 58, 59), are lacking. Therefore, the data strongly support a piston-type mode of TM signaling by the TM2 homodimer from the periplasmic α6-PAS_P_ to the linker on the cytoplasmic side of the membrane.

The linker (C_199_ILVKVLKKILFG_211_) connecting TM2 with PAS_C_ comprises three positively charged Lys residues and an LxxxLxxxL sequence. The linker is α-helical similar to TM2, but differs from TM2 in the extent of helix dimerization, the response to fumarate, and helical spacing. In the linker, the CL efficiency responded to the presence of fumarate (Fig. 6*B*), implying structural changes upon fumarate addition. The impact of fumarate differed between the N- and C-terminal parts of the linker; the response of Ile208 and Leu209 in the C terminus was different from that of residues Leu201 and Leu205 in the N terminus (Fig. 4*B*). This suggests that fumarate-induced signaling involves complex structural rearrangements of the linker, including attenuated helix interactions or separation of the helical region in the presence of fumarate. Thus, the linker is predicted to convert the piston movement of TM2 into structural rearrangements and to control PAS_C_ structure and activity.

**Figure 7:**
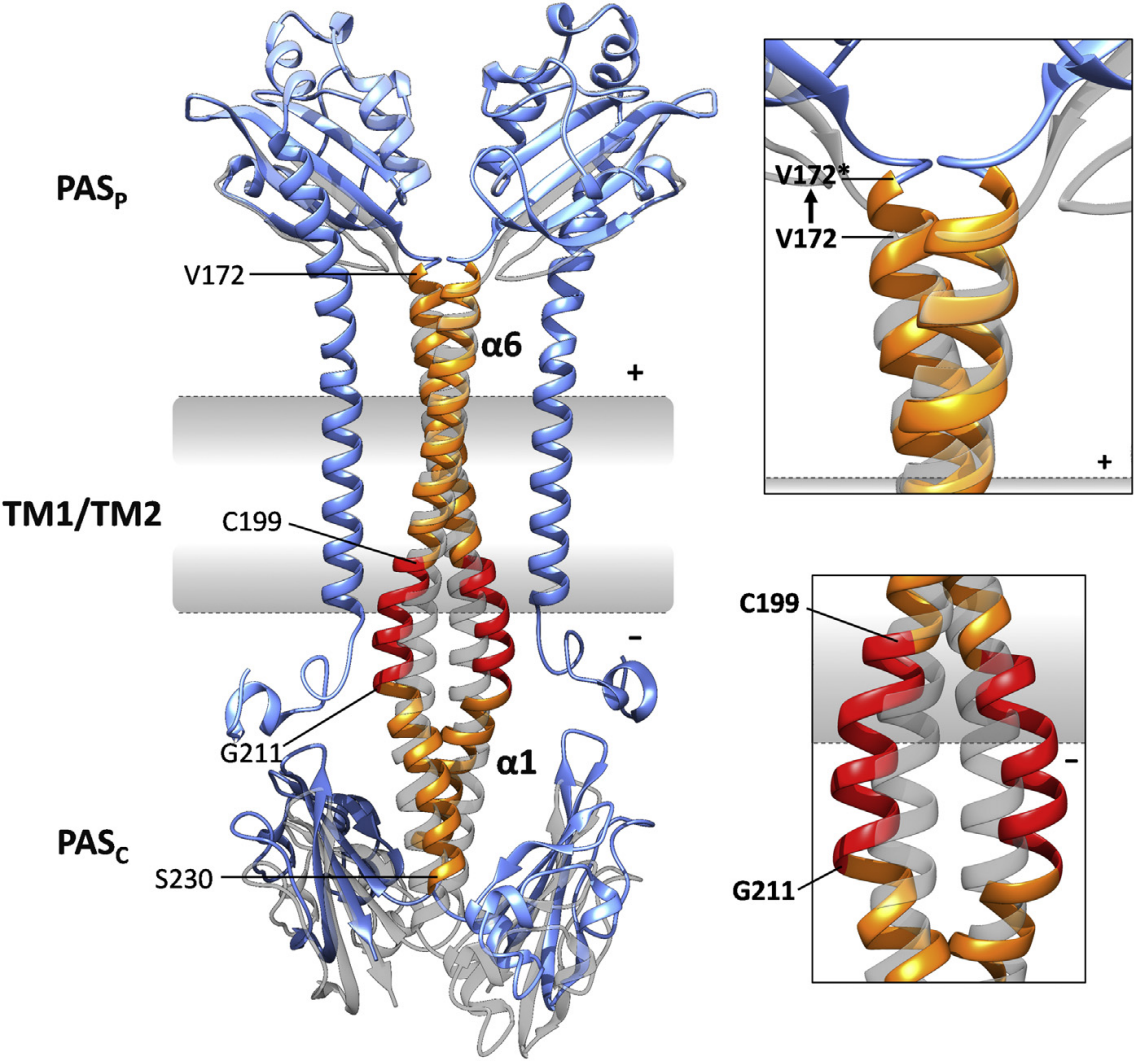
**Model of dimeric DcuS with an emphasis on the bihelical TM2 region extending from α6 of PAS**_**P**_**into α1 of PAS**_**C**_**, and its role in TM signaling.** Dimeric DcuS with the homodimeric α6(PAS_P_)-TM2-linker-α1(PAS_C_) helix in the presence (*orange* or *red*) or absence (*gray*) of fumarate. α6, α1, and amino acid residues delineating domains are shown: Val172 as the start site for α6, Val172/Cys199 as the limits of the rigid α6(PAS_C_)-TM2 helix, Cys199/Gly211 as the linker region, and Gly211/Ser230 as α1. The structures of PAS_P_, PAS_C_, and TM1 are shown in *light blue*. The piston movement of TM2 upon fumarate activation (18) is presented in the top magnification box by the movement of residue Val172 (V172) to the periplasmic position (V172∗) in the fumarate-activated state. Val172 marks the starting point of the transient α-helix, and Cys199 and Gly211 the approximate starting and end points of the linker. The supposed movement of the linker monomers upon fumarate activation is shown in the bottom magnification box. A simplified scheme presenting the structural elements of DcuS and their structural rearrangements is shown in Figure S10. DcuS was modeled as a composition of the structures of PAS_P_, PAS_C_, and modeled TM2 and the linker from CL studies. UCSF Chimera was used to fuse the structures obtained by homology modeling (PAS_P_, PAS_C_) and predicted structures (TM2, linker). The structure of the apo PAS_P_ monomer was obtained using the structure of DcuS homolog CitA as a template (6, PDB ID:2V9A). The PAS_C_ dimer structure was modeled using the structure of CitA as a template (PDB ID: 5FQ1). The helix dimer from Val172 to Glu213 was derived by structural analysis of the C-terminal region of PAS_P_ (α6) and the N-terminal region of PAS_C_ (α1) (3, 9, 34), and the CL data (18, this work) for V172 to E213. The cytoplasmic N-terminal coil, TM1, and the TM2-PAS_C_ linker were predicted as single subdomains by the server I-TASSER (60). The kinase domain is not shown.

In DcuS, the basis for TM signaling is the movement of a piston (TM2) that crosses the complete membrane as a stable homodimer (Fig. 7). Dimerization of TM2 is central to signaling and maintained in the fumarate-activated and the inactive state. In the closely homologous CitA, movement of the piston is triggered by the binding of citrate to the PAS_P_ domain. Compaction of the binding site then drives an uplift of helix α6-PAS_P_ that directly runs into TM2 (6, 21). By analogy to CitA, a similar scenario has been suggested for DcuS (6, 61) to trigger the piston movement of TM2. On the cytoplasmic side, the TM2 piston movement is perceived by the linker. In the linker the α-helical structure is modified in stability and +3/+4 spacing of interacting residues in response to fumarate availability. Thus, the mode of signal transduction likely changes in this region from piston mode to reorganization (or destabilization) of the helices in response to fumarate binding (Figs. 7 and S10). The structural changes in the linker are supposed to subsequently reorganize the PAS_C_ dimer, which then controls the kinase domain and activity as suggested previously (9, 10). Sensor kinase BvgS from *B. pertussis* is also composed of a periplasmic signal receiver domain followed by a TM helix and a cytoplasmic 28 aa linker connecting TM2 and a PAS domain. BvgS shows a comparable mechanism in TM signaling by TM2 and signal conversion of the linker by helical coiled-coil disruption (62). Piston-type displacement of BvgS TM2, however, is small, and TM2 is lacking a GxxxG motif.

For other sensor kinases, TM signaling involving a piston-type shift in helices has been shown or suggested, though its importance appears to be less in the entire mechanism. Remarkably, a GxxxG motif stabilizing TM helix dimers is apparently missing in other sensor kinases that also contain two TM helices for TM signaling, such as NarQ, PhoQ, or even the closely related CitA (Fig. S1). Therefore the S_186_xxxGxxxG_194_ motif, as important as it is for DcuS, it is apparently not widespread in sensor kinases with piston-mode TM signaling. TM signaling by DcuS resembles, in some aspects, that of the nitrate sensor NarQ. The homodimeric NarQ contains an antiparallel four-stranded coiled-coil structure in the membrane, linking the periplasmic sensor to a cytoplasmic HAMP domain (7). Nitrate binding displaces the sensory domain and shifts the TM1 helices to the periplasmic side relative to TM2. However, a Pro-kink in the C-terminal part of TM2 is crucial for transferring the conformational rearrangement to the HAMP domain in a lever-like mechanism (7). Overall, nitrate binding causes helical rotation and diagonal scissoring of the α-helices connected to the sensor domain, which then leads to a piston-like motion in the TM region (31), demonstrating that TM signaling may apply a combination of conformational changes for signal transduction. The PhoQ-PhoP two-component system of enteric bacteria senses divalent cations and antimicrobial peptides (63, 64). Signal transfer by the PhoQ kinase involves scissor-like diagonal displacement of TM helices, or a combination of scissoring and rotational movements, which is suggested by Cys-scanning disulfide CL and modeling (3, 14, 16). In the distantly related sensory rhodopsin from *Natronobacterium pharaonis*, light excitation induces a rotary motion of the second TM helix that controls finally kinase activity (65). It appears therefore that membrane-bound sensor kinases apply various types of conformational changes for transmembrane signaling, and various modes can be combined.

## Experimental procedures

### Bacterial growth and molecular genetics

The *E. coli*, K12 derivatives, plasmids, and primers are listed in Tables S1 and S2. Molecular genetic techniques were performed according to standard procedures (66). The plasmids encoding the GALLEX constructs were generated by inserting TM1 and TM2 sequences obtained by PCR from pMW181 into pBLM100 *via* the *Sac*I and *Spe*I sites. For all Cys CL experiments, cells were grown aerobically in 700 μl LB medium (with or without 50 mM sodium fumarate) and expression of *dcuS* induced after 2 h with 1 mM IPTG (18). For the *dcuB-lacZ* reporter gene assays, the cells were cultivated anaerobically to an OD_578_ of 0.5–0.8 in enriched mineral medium (eM9) containing glycerol (50 mM) and dimethyl sulfoxide (DMSO; 20 mM) (18, 30). Sodium fumarate (20 mM) was used as an effector as indicated. For BACTH assays, bacteria were grown in 48-well plates with vigorous shaking to an OD_578_ of 0.5–0.8 (18). To measure interactions with the GALLEX system, freshly transformed cells were used to inoculate 5 ml of overnight culture in LB medium. The next day, 5 ml medium was inoculated with 0.1 ml of the overnight cultures and grown aerobically to an OD_578_ of 0.6 in the presence of ampicillin and 5 μM IPTG. β-Galactosidase activity was measured as described elsewhere ([10, 67]; supplemental material). For the *dcuB-lacZ* reporter gene assay, strain IMW260 was transformed with derivatives of pMW181 (DcuS Wt). To test the BACTH (49, 50), *E. coli* BTH101 was cotransformed with pairs of plasmids encoding DcuS with N-terminal fusions of T18 and T25 fragments (68). In the GALLEX TM-dimerization assay (51), *E coli* SU101 was transformed with plasmids encoding the GpA and DcuS TM2 fusion proteins.

### Oxidative *in vivo* cysteine cross-linking

To test Cys CL (18), *E. coli* C43(DE3) was transformed with single-Cys variants encoded by pMW336. Bacteria (OD_578_ of 2.5–3.0) were treated for the standard procedure with Cu(II)-1,10-phenanthroline as described (18). The reaction was stopped with 12.5 mM EDTA and 12.5 mM *N*-ethylmaleimide, and the bacteria evaluated by SDS-PAGE under oxidative conditions (18). For time-resolved CL, the reaction was terminated after 0.5, 3, 10, and 20 min. Oxidant concentrations and temperatures were adjusted in the following way: 0.5 mM CuSO_4_, 1.625 mM 1,10-phenanthroline, and 4 °C for variants S186C, G194C, L208C, and L209C; 2 mM CuSO_4_, 7.5 mM 1,10-phenanthroline, and 4 °C for G190C; 4 mM CuSO_4_, 13 mM 1,10-phenanthroline, and 4 °C for L205C; 4 mM CuSO_4_, 13 mM 1,10-phenanthroline, and 20 °C for L201C (all concentrations refer to the reaction mixture). For quantitative analysis of CL efficiency, the X-ray films from the western blots were scanned to obtain a digital image. In the image processing software ImageJ (69), the scanned images were converted to black and white. The individual lanes of each Cys variant per X-ray film were marked by identical squares. The band size and density were plotted as integrals by ImageJ. The relative integral values were measured using the Wand (tracing) Tool. These values were used for calculating the ratio of the CL product to total DcuS.

### DcuS expression and membrane localization

Bacteria (100 or 200 μg cell protein) were sedimented, dissolved in 2× SDS-PAGE sample buffer, and applied to polyacrylamide gels. For oxidative Cys-CL, dithiothreitol was omitted from buffers. For SDS-PAGE (70), 12% gels were used; for Cys CL experiments, gradient gels (5–12% acrylamide) were used. Western blotting was performed (18) with rabbit polyclonal antiserum raised against PAS_P_ of DcuS and peroxidase-coupled goat anti-rabbit-IgG secondary antibody. To test GALLEX constructs, rabbit anti-MBP antiserum (New England Biolabs) was used. Bands were visualized using WesternBright ECL/peroxide and X-ray films (Advansta). The DcuS bands were quantified by measuring the area of the band and the staining intensities in scanned blots. Quantitative evaluation was performed using ImageJ software (69).

## Data availability

Data are contained within the article or the Supporting information. Quantitative data from western blotting (ImageJ) are available from the corresponding author on request.

## Conflict of interest

The authors declare that they have no conflicts of interest with the contents of this article.

## References

[1] Mascher T., Helmann J.D., Unden G. (2006). Stimulus perception in bacterial signal-transducing histidine kinases. Microbiol. Mol. Biol. Rev..

[2] Krell T., Lacal J., Busch A., Silva-Jiménez H., Guazzaroni M.-E., Ramos J.L. (2010). Bacterial sensor kinases: diversity in the recognition of environmental signals. Annu. Rev. Microbiol..

[3] Cheung J., Hendrickson W.A. (2008). Crystal structures of C4-dicarboxylate ligand complexes with sensor domains of histidine kinases DcuS and DctB. J. Biol. Chem..

[4] Cheung J., Hendrickson W.A. (2009). Structural analysis of ligand stimulation of the histidine kinase NarX. Structure.

[5] Cheung J., Hendrickson W.A. (2010). Sensor domains of two-component regulatory systems. Curr. Opin. Microbiol..

[6] Sevvana M., Vijayan V., Zweckstetter M., Reinelt S., Madden D.R., Herbst-Irmer R., Sheldrick G.M., Bott M., Griesinger C., Becker S. (2008). A ligand-induced switch in the periplasmic domain of sensor histidine kinase CitA. J. Mol. Biol..

[7] Gushchin I., Melnikov I., Polovinkin V., Ishchenko A., Yuzhakova A., Buslaev P., Bourenkov G., Grudinin S., Round E., Balandin T., Borshchevskiy V., Willbold D., Leonard G., Büldt G., Popov A. (2017). Mechanism of transmembrane signaling by sensor histidine kinases. Science.

[8] Hulko M., Berndt F., Gruber M., Linder J.U., Truffault V., Schultz A., Martin J., Schultz J.E., Lupas A.N., Coles M. (2006). The HAMP domain structure implies helix rotation in transmembrane signaling. Cell.

[9] Etzkorn M., Kneuper H., Dünnwald P., Vijayan V., Krämer J., Griesinger C., Becker S., Unden G., Baldus M. (2008). Plasticity of the PAS domain and a potential role for signal transduction in the histidine kinase DcuS. Nat. Struct. Mol. Biol..

[10] Monzel C., Degreif-Dünnwald P., Gröpper C., Griesinger C., Unden G. (2013). The cytoplasmic PASC domain of the sensor kinase DcuS of Escherichia coli: role in signal transduction, dimer formation, and DctA interaction. Microbiologyopen.

[11] Marina A., Waldburger C.D., Hendrickson W.A. (2005). Structure of the entire cytoplasmic portion of a sensor histidine-kinase protein. EMBO J..

[12] Wang C., Sang J., Wang J., Su M., Downey J.S., Wu Q., Wang S., Cai Y., Xu X., Wu J., Senadheera D.B., Cvitkovitch D.G., Chen L., Goodman S.D., Han A. (2013). Mechanistic insights revealed by the crystal structure of a histidine kinase with signal transducer and sensor domains. PLoS Biol..

[13] Casino P., Rubio V., Marina A. (2010). The mechanism of signal transduction by two-component systems. Curr. Opin. Struct. Biol..

[14] Lemmin T., Soto C.S., Clinthorne G., DeGrado W.F., Dal Peraro M. (2013). Assembly of the transmembrane domain of E. coli PhoQ histidine kinase: implications for signal transduction from molecular simulations. PLoS Comput. Biol..

[15] Diensthuber R.P., Bommer M., Gleichmann T., Möglich A. (2013). Full-length structure of a sensor histidine kinase pinpoints coaxial coiled coils as signal transducers and modulators. Structure.

[16] Molnar K.S., Bonomi M., Pellarin R., Clinthorne G.D., Gonzalez G., Goldberg S.D., Goulian M., Sali A., DeGrado W.F. (2014). Cys-scanning disulfide crosslinking and bayesian modeling probe the transmembrane signaling mechanism of the histidine kinase, PhoQ. Structure.

[17] Falke J.J. (2014). Piston versus scissors: chemotaxis receptors versus sensor His-kinase receptors in two-component signaling pathways. Structure.

[18] Monzel C., Unden G. (2015). Transmembrane signaling in the sensor kinase DcuS of Escherichia coli: a long-range piston-type displacement of transmembrane helix 2. Proc. Natl. Acad. Sci. U. S. A..

[19] Bhate M.P., Molnar K.S., Goulian M., DeGrado W.F. (2015). Signal transduction in histidine kinases: insights from new structures. Structure.

[20] Zschiedrich C.P., Keidel V., Szurmant H. (2016). Molecular mechanisms of two-component signal transduction. J. Mol. Biol..

[21] Salvi M., Schomburg B., Giller K., Graf S., Unden G., Becker S., Lange A., Griesinger C. (2017). Sensory domain contraction in histidine kinase CitA triggers transmembrane signaling in the membrane-bound sensor. Proc. Natl. Acad. Sci. U. S. A..

[22] Zientz E., Bongaerts J., Unden G. (1998). Fumarate regulation of gene expression in Escherichia coli by the DcuSR (dcuSR genes) two-component regulatory system. J. Bacteriol..

[23] Scheu P.D., Kim O.B., Griesinger C., Unden G. (2010). Sensing by the membrane-bound sensor kinase DcuS: exogenous versus endogenous sensing of C(4)-dicarboxylates in bacteria. Future Microbiol..

[24] Kleefeld A., Ackermann B., Bauer J., Krämer J., Unden G. (2009). The fumarate/succinate antiporter DcuB of Escherichia coli is a bifunctional protein with sites for regulation of DcuS-dependent gene expression. J. Biol. Chem..

[25] Witan J., Bauer J., Wittig I., Steinmetz P.A., Erker W., Unden G. (2012). Interaction of the Escherichia coli transporter DctA with the sensor kinase DcuS: presence of functional DctA/DcuS sensor units. Mol. Microbiol..

[26] Unden G., Strecker A., Kleefeld A., Kim O.B. (2016). C4-Dicarboxylate utilization in aerobic and anaerobic growth. EcoSal Plus.

[27] Scheu P.D., Liao Y.-F., Bauer J., Kneuper H., Basché T., Unden G., Erker W. (2010). Oligomeric sensor kinase DcuS in the membrane of Escherichia coli and in proteoliposomes: chemical cross-linking and FRET spectroscopy. J. Bacteriol..

[28] Kneuper H., Janausch I.G., Vijayan V., Zweckstetter M., Bock V., Griesinger C., Unden G. (2005). The nature of the stimulus and of the fumarate binding site of the fumarate sensor DcuS of Escherichia coli. J. Biol. Chem..

[29] Pappalardo L., Janausch I.G., Vijayan V., Zientz E., Junker J., Peti W., Zweckstetter M., Unden G., Griesinger C. (2003). The NMR structure of the sensory domain of the membranous two-component fumarate sensor (histidine protein kinase) DcuS of Escherichia coli. J. Biol. Chem..

[30] Krämer J., Fischer J.D., Zientz E., Vijayan V., Griesinger C., Lupas A., Unden G. (2007). Citrate sensing by the C4-dicarboxylate/citrate sensor kinase DcuS of Escherichia coli: binding site and conversion of DcuS to a C4-dicarboxylate- or citrate-specific sensor. J. Bacteriol..

[31] Gushchin I., Orekhov P., Melnikov I., Polovinkin V., Yuzhakova A., Gordeliy V. (2020). Sensor histidine kinase NarQ activates via helical rotation, diagonal scissoring, and eventually piston-like shifts. Int. J. Mol. Sci..

[32] Lee G.F., Lebert M.R., Lilly A.A., Hazelbauer G.L. (1995). Transmembrane signaling characterized in bacterial chemoreceptors by using sulfhydryl cross-linking *in vivo*. Proc. Natl. Acad. Sci. U. S. A..

[33] Hughson A.G., Hazelbauer G.L. (1996). Detecting the conformational change of transmembrane signaling in a bacterial chemoreceptor by measuring effects on disulfide cross-linking *in vivo*. Proc. Natl. Acad. Sci. U. S. A..

[34] Weisenburger S., Boening D., Schomburg B., Giller K., Becker S., Griesinger C., Sandoghdar V. (2017). Cryogenic optical localization provides 3D protein structure data with Angstrom resolution. Nat. Methods.

[35] Janausch I.G., Garcia-Moreno I., Unden G. (2002). Function of DcuS from Escherichia coli as a fumarate-stimulated histidine protein kinase *in vitro*. J. Biol. Chem..

[36] Senes A., Gerstein M., Engelman D.M. (2000). Statistical analysis of amino acid patterns in transmembrane helices: the GxxxG motif occurs frequently and in association with beta-branched residues at neighboring positions. J. Mol. Biol..

[37] Kleiger G., Grothe R., Mallick P., Eisenberg D. (2002). GXXXG and AXXXA: common alpha-helical interaction motifs in proteins, particularly in extremophiles. Biochemistry.

[38] Dawson J.P., Weinger J.S., Engelman D.M. (2002). Motifs of serine and threonine can drive association of transmembrane helices. J. Mol. Biol..

[39] Schneider D., Engelman D.M. (2004). Motifs of two small residues can assist but are not sufficient to mediate transmembrane helix interactions. J. Mol. Biol..

[40] Kim S., Jeon T.-J., Oberai A., Yang D., Schmidt J.J., Bowie J.U. (2005). Transmembrane glycine zippers: physiological and pathological roles in membrane proteins. Proc. Natl. Acad. Sci. U. S. A..

[41] Bailey T.L., Williams N., Misleh C., Li W.W. (2006). MEME: discovering and analyzing DNA and protein sequence motifs. Nucleic Acids Res..

[42] Huerta-Cepas J., Szklarczyk D., Forslund K., Cook H., Heller D., Walter M.C., Rattei T., Mende D.R., Sunagawa S., Kuhn M., Jensen L.J., Mering C. von, Bork P. (2016). eggNOG 4.5: a hierarchical orthology framework with improved functional annotations for eukaryotic, prokaryotic and viral sequences. Nucleic Acids Res..

[43] Lemmon M.A., Flanagan J.M., Treutlein H.R., Zhang J., Engelman D.M. (1992). Sequence specificity in the dimerization of transmembrane alpha-helices. Biochemistry.

[44] Fleming K.G., Engelman D.M. (2001). Specificity in transmembrane helix-helix interactions can define a hierarchy of stability for sequence variants. Proc. Natl. Acad. Sci. U. S. A..

[45] Miroux B., Walker J.E. (1996). Over-production of proteins in Escherichia coli: mutant hosts that allow synthesis of some membrane proteins and globular proteins at high levels. J. Mol. Biol..

[46] Yanisch-Perron C., Vieira J., Messing J. (1985). Improved M13 phage cloning vectors and host strains: nucleotide sequences of the M13mpl8 and pUC19 vectors. Gene.

[47] Treptow N.A., Shuman H.A. (1985). Genetic evidence for substrate and periplasmic-binding-protein recognition by the MalF and MalG proteins, cytoplasmic membrane components of the Escherichia coli maltose transport system. J. Bacteriol..

[48] Karimova G., Ullmann A., Ladant D. (2001). Protein-protein interaction between Bacillus stearothermophilus tyrosyl-tRNA synthetase subdomains revealed by a bacterial two-hybrid system. J. Mol. Microbiol. Biotechnol..

[49] Karimova G., Pidoux J., Ullmann A., Ladant D. (1998). A bacterial two-hybrid system based on a reconstituted signal transduction pathway. Proc. Natl. Acad. Sci. U. S. A..

[50] Karimova G., Ullmann A., Ladant D. (2000). Bordetella pertussis adenylate cyclase toxin as a tool to analyze molecular interactions in a bacterial two-hybrid system. Int. J. Med. Microbiol..

[51] Schneider D., Engelman D.M. (2003). GALLEX, a measurement of heterologous association of transmembrane helices in a biological membrane. J. Biol. Chem..

[52] Cymer F., Veerappan A., Schneider D. (2012). Transmembrane helix-helix interactions are modulated by the sequence context and by lipid bilayer properties. Biochim. Biophys. Acta.

[53] Dmitrova M., Younès-Cauet G., Oertel-Buchheit P., Porte D., Schnarr M., Granger-Schnarr M. (1998). A new LexA-based genetic system for monitoring and analyzing protein heterodimerization in Escherichia coli. Mol. Gen. Genet..

[54] Polyansky A.A., Chugunov A.O., Volynsky P.E., Krylov N.A., Nolde D.E., Efremov R.G. (2014). PREDDIMER: a web server for prediction of transmembrane helical dimers. Bioinformatics.

[55] Plevin M.J., Mills M.M., Ikura M. (2005). The LxxLL motif: a multifunctional binding sequence in transcriptional regulation. Trends Biochem. Sci..

[56] Heijne G. von (2006). Membrane-protein topology. Nat. Rev. Mol. Cell Biol..

[57] Kitanovic S., Ames P., Parkinson J.S. (2011). Mutational analysis of the control cable that mediates transmembrane signaling in the Escherichia coli serine chemoreceptor. J. Bacteriol..

[58] Senes A., Engel D.E., DeGrado W.F. (2004). Folding of helical membrane proteins: the role of polar, GxxxG-like and proline motifs. Curr. Opin. Struct. Biol..

[59] Riek R.P., Rigoutsos I., Novotny J., Graham R.M. (2001). Non-alpha-helical elements modulate polytopic membrane protein architecture. J. Mol. Biol..

[60] Yang J., Zhang Y. (2015). I-TASSER server: new development for protein structure and function predictions. Nucleic Acids Res..

[61] Kneuper H., Scheu P.D., Etzkorn M., Sevvana M., Dünnwald P., Becker S., Baldus M., Griesinger C., Unden G., Spiro S., Dixon R. (2010). Sensing ligands by periplasmic sensing histidine kinases with sensory PAS domains. Sensory Mechanisms in Bacteria: Molecular Aspects of Signal Recognition.

[62] Lesne E., Dupré E., Locht C., Antoine R., Jacob-Dubuisson F. (2017). Conformational changes of an interdomain linker mediate mechanical signal transmission in sensor kinase BvgS. J. Bacteriol..

[63] Bader M.W., Sanowar S., Daley M.E., Schneider A.R., Cho U., Xu W., Klevit R.E., Le Moual H., Miller S.I. (2005). Recognition of antimicrobial peptides by a bacterial sensor kinase. Cell.

[64] Cho U.S., Bader M.W., Amaya M.F., Daley M.E., Klevit R.E., Miller S.I., Xu W. (2006). Metal bridges between the PhoQ sensor domain and the membrane regulate transmembrane signaling. J. Mol. Biol..

[65] Wegener A.A., Klare J.P., Engelhard M., Steinhoff H.J. (2001). Structural insights into the early steps of receptor-transducer signal transfer in archaeal phototaxis. EMBO J..

[66] Sambrook J., Russell D.W. (2001). Molecular Cloning: A Laboratory Manual.

[67] Miller J.H. (1992). A Short Course in Bacterial Genetics: A Laboratory Manual and Handbook for Escherichia coli and Related Bacteria.

[68] Scheu P.D., Witan J., Rauschmeier M., Graf S., Liao Y.-F., Ebert-Jung A., Basché T., Erker W., Unden G. (2012). CitA/CitB two-component system regulating citrate fermentation in Escherichia coli and its relation to the DcuS/DcuR system *in vivo*. J. Bacteriol..

[69] Schneider C.A., Rasband W.S., Eliceiri K.W. (2012). NIH Image to ImageJ: 25 years of image analysis. Nat. Methods.

[70] Laemmli U.K. (1970). Cleavage of structural proteins during the assembly of the head of bacteriophage T4. Nature.

