# Supplementary material for: Transmembrane signaling and cytoplasmic signal conversion by dimeric transmembrane helix 2 and a linker domain of the DcuS sensor kinase
Source: J Biol Chem. 2020 Dec 10;296:100148. doi: 10.1074/jbc.RA120.015999 (PMC7857512; doi:10.1074/jbc.RA120.015999)
Supplement: Figures S1 to S10 and Tables S1 and S2 [file mmc1.docx]

**Transmembrane signaling and cytoplasmic signal conversion by dimeric transmembrane helix 2 and a linker domain of the DcuS sensor kinase**

Marius Stopp^1^, Philipp Aloysius Steinmetz^1^, Christopher Schubert^1^, Christian Griesinger^2^. Dirk Schneider^3^, Gottfried Unden^1^

^1^Microbiology and Wine Research, Institute for Molecular Physiology, Johannes Gutenberg University Mainz, Mainz, Germany

^2^Department of NMR-based Structural Biology, Max Planck Institute for Biophysical Chemistry, 37077 Gottingen, Germany

^3^, Department of Chemistry, Biochemistry, Johannes Gutenberg University Mainz, Mainz, Germany

Address correspondence to:

Gottfried Unden,

Microbiology and Wine Research, Institute for Molecular Physiology; University of Mainz,

Johann-Joachim-Becher-Weg 15
55099 Mainz, Germany

Phone: +49-6131-3923550

unden@uni-mainz.de

**Figure S1. Detection of DcuS in SDS-PAGE and Western blotting.** (A) SDS-PAGE gel Coomassie PAGE Blue staining. (B) Ponceau S staining of a nitro-cellulose membrane after semi-dry Western blotting. (C) DcuS chemiluminescence detection on X-ray film with an antiserum raised against PAS_P_ of DcuS. [1] PageRuler Prestained Protein Ladder (Thermo Scientific). The numbers on the right represent molecular weight [kDa]; [2] 2 µg purified DcuS [3] 0.5 µg purified DcuS. Overproduction and purification of DcuS for [1] and [2] was performed as described in (35). [4] DcuS G190C, untreated; [5] DcuS G190C, crosslinked. *In vivo* CL and sample preparation for [3] and [4] was performed as described in Methods.


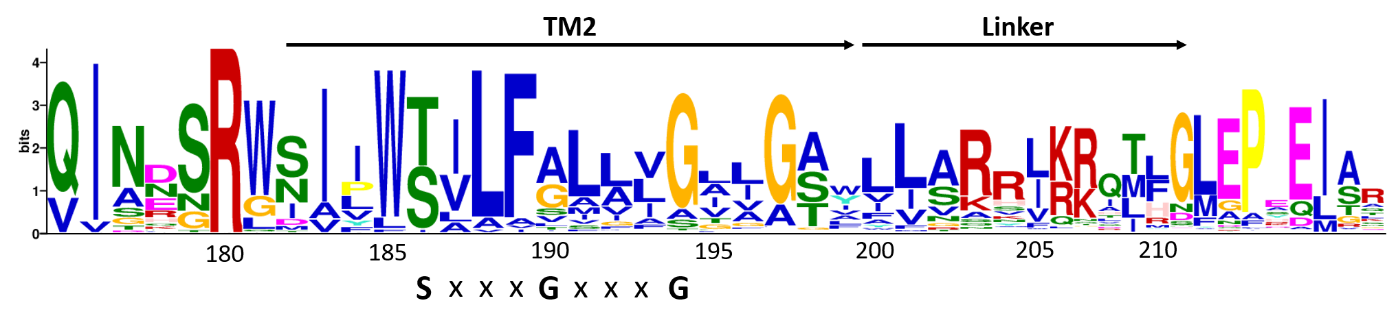


**Figure S2. Sequence conservation in the TM2-PAS_C_ linker region of DcuS and CitA proteins.** The sequence logo was generated with MEME Suite version 5.0.5. (41) derived from the sequence of 654 DcuS and CitA orthologous proteins obtained from EggNOG database version 4.5.1 (42). The arrows on top of the logo indicate positions of the TM2 and of the Linker domains of DcuS from *E. coli*. The numbers below the logo represent corresponding positions in DcuS of *E. coli* and the SxxxGxxxG sequence motif.


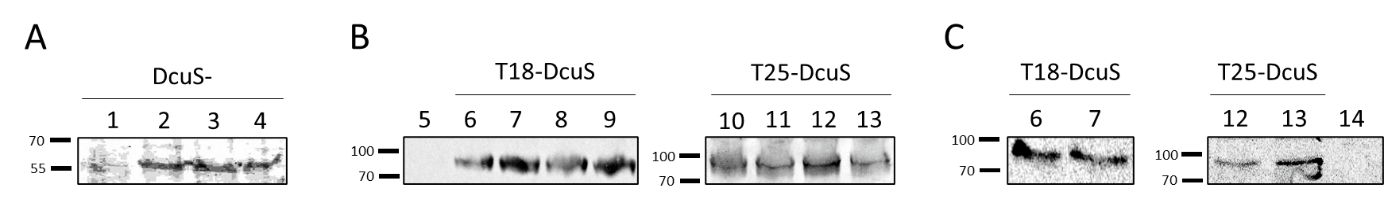


**Figure S3. Levels of DcuS and TM2 variants and membrane integration detected by SDS-PAGE and Western blotting with anti-DcuS (PASP).** (A) DcuS produced from plasmids for the *dcuB-lacZ* assay of Fig. 2. Cells were cultivated for the assay and whole cells (200 μg protein) were applied to SDS-PAGE. [1] DcuS - (IMW260); [2] DcuS Wt (pMW181); [3] DcuS(G190A+G194A) (pMW813); [4] DcuS(S186C+G190A) (pMW809). (B) Bacteria for DcuS BACTH testing. The type of fusion (T18 or T25) fused is indicated above the figure. Cells were cultivated as for the BACTH assay and bacteria (200 μg cell protein) was applied to SDS-PAGE. [5] *E. coli* BTH101; [6] *E. coli* BTH101 with pMW429 producing T18-DcuS; [7] *E. coli* BTH101 with pMW1212 producing T18-DcuS(G190A+G194A); [8] *E. coli* BTH101 with pMW1210 producing T18-DcuS(S186C+G190A); [9] *E. coli* BTH101 with pMW1755 producing T18-DcuS(G194A); [10] *E. coli* BTH101 with pMW1751 producing T25-DcuS(G194A); [11] *E. coli* BTH101 with pMW1209 producing T25-DcuS(S186C+G190A); [12] *E. coli* BTH101 with pMW1211 producing T25-DcuS(G190A+G194A); [13] *E. coli* BTH101 with pMW426 producing T25-DcuS. (C) Membrane localization of DcuS BACTH fusion proteins. *E. coli* JM109 was cultivated as for the BACTH assay. Bacteria were pelleted and resuspended in buffer (50 mM Tris/HCl at pH 7.7, 10 mM MgCl2). Bacteria were then disrupted with glass beads (0.1 mm) by FastPrep-24 (6.5 m/s, 6x1 min). Debris was separated by centrifugation and the membrane fraction was sedimented by centrifugation (150,000×*g* for 65 min 4°C). 400 μg protein of the membranes was loaded on SDS-gels. [6] *E. coli* JM109 with pMW429 producing T18-DcuS; [7] *E. coli* JM109 with pMW1212 producing T18-DcuS(G190A+G194A); [12] *E. coli* JM109 with pMW1211 producing T25-DcuS(G190A/G194A); [13] *E. coli* JM109 with pMW426 producing T25-DcuS Wt (pMW426; [14] *E. coli* JM109.

**Figure S4. Validation of TM2 GALLEX constructs for optimal length.** On top of the plot the respective selected sequences of the TM2 constructs of varying length, and the corresponding length of amino acid residues are shown for TM2 in parantheses below the graph. The constructs TM_GpA [17]_ Wt and TM_GpA [17]_ G83I served as controls for interaction in the GALLEX system (51). The bars represent the arithmetic mean of three biological replicates and the positive SD is shown.


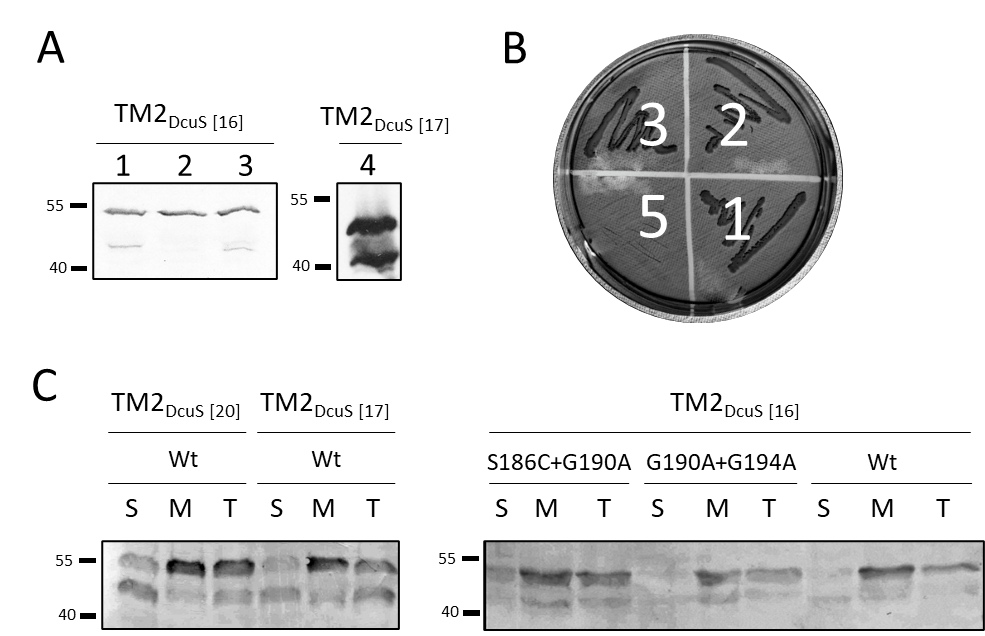


**Figure S5. Expression of TM2 GALLEX constructs (53 to 55 kDa depending on construct) and membrane localization.** (A) Expression of TM2 GALLEX fusions. Strain and growth conditions were the same as for the GALLEX assay. Bacteria (strain NT326) (200 μg protein) were used for SDS-PAGE and TM2 GALLEX fusions were detected by Western blotting with anti-MBP [1] *E. coli* NT326 with pMW1963 producing TM2DcuS[16] (Wt); [2] *E. coli* NT326 with pMW1964 producing TM2DcuS[16](G190A+G194A); [3] *E. coli* NT326 with pMW1965 producing TM2DcuS[16](S186C+G190A); [4] *E. coli* NT326 with pMW1961 producing TM2DcuS[17] (Wt). (B) TM2 GALLEX fusion constructs growth complementation to test membrane insertion and topology of the TM2 construct with a chain length of 16 amino acid residues (compare Fig. S4). The plasmids were transformed into maltose binding protein deficient *E. coli* NT326 and tested for growth on minimal medium agar plates with 0.4 % (w/v) maltose at 37°C for 72 h (45). [1] NT326 with pMW1963 encoding TM2DcuS [16]; [2] NT326 with pMW1964 encoding TM2DcuS[16](G190A+G194A); [3] NT326 with pMW1965 encoding TM2DcuS[16](S186C+G190A); [5] NT326. (C) Membrane localization of TM2 GALLEX fusion constructs. Growth conditions were the same as for the GALLEX assay but expression was carried out in *E. coli* NT326. Membrane preparation was as for Fig. S3. DcuS TM2 GALLEX fusions were detected by Western blotting with anti-MBP. The corresponding TM2 fusion construct is shown above the images. [S] soluble fraction; [M] membrane; [T] total protein.

**Figure S6. Effect of Ala substitutions in the TM2-PAS_C_ linker region on *dcuB-lacZ* expression.** Expression of *dcuB* and effect of the substitutions was tested in the *dcuS* negative *E. coli* strain IMW260 (DcuS -) complemented with plasmid (pMW181)-encoded Wt DcuS (DcuS Wt) and derivatives of DcuS with Ala substitutions in the TM2-PAS_C_ linker (Cys199 to Gly211). Cells were cultivated under anaerobic conditions in eM9 medium with glycerol plus 20 mM DMSO with or without 20 mM di-sodium fumarate to mid-exponential growth and β-galactosidase activities were measured. All activities were normalized to the Wt control of DcuS in the fumarate activated state. Three biological replicates were tested and the SD is shown.


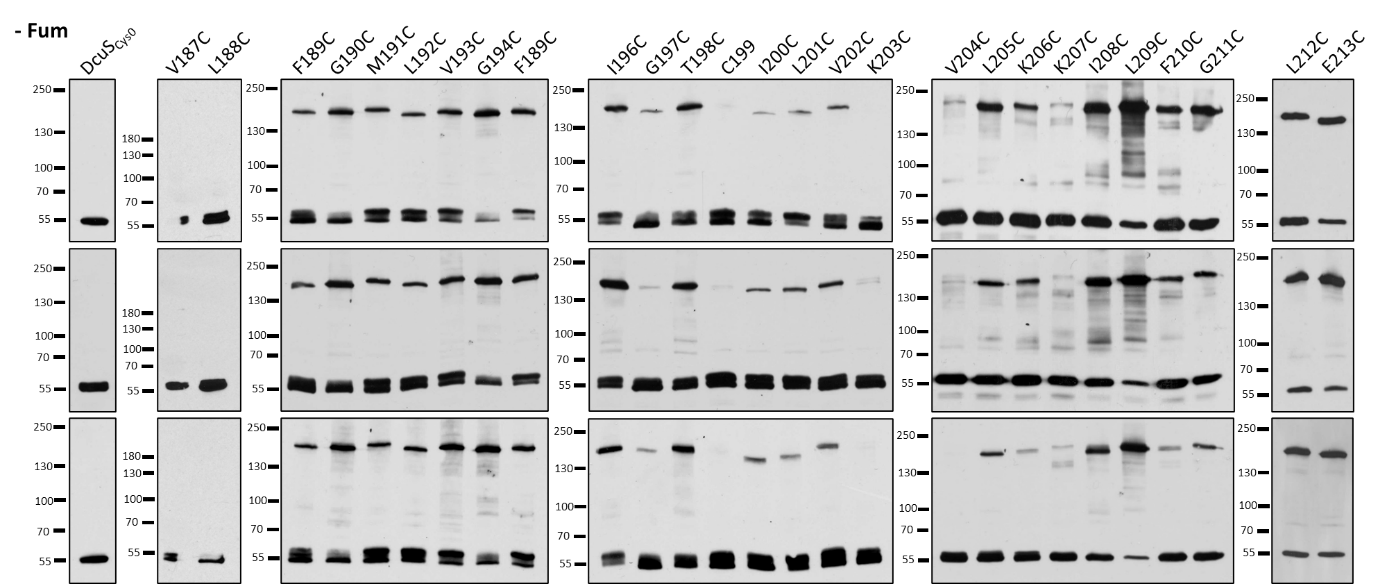


**Figure S7. Detection of DcuS after cross-linking of bacteria with Cu^2+^ phenanthroline in the absence of fumarate (SDS-PAGE and Western blot with anti-DcuS (PAS_P_).** Cell protein was separated in non reducing SDS-PAGE and DcuS and CL-products were detected by chemiluminescence after Western blotting as described (18). Scans of the X-ray films are shown. Each experiment was performed in biological triplicates that are arranged one below each other.


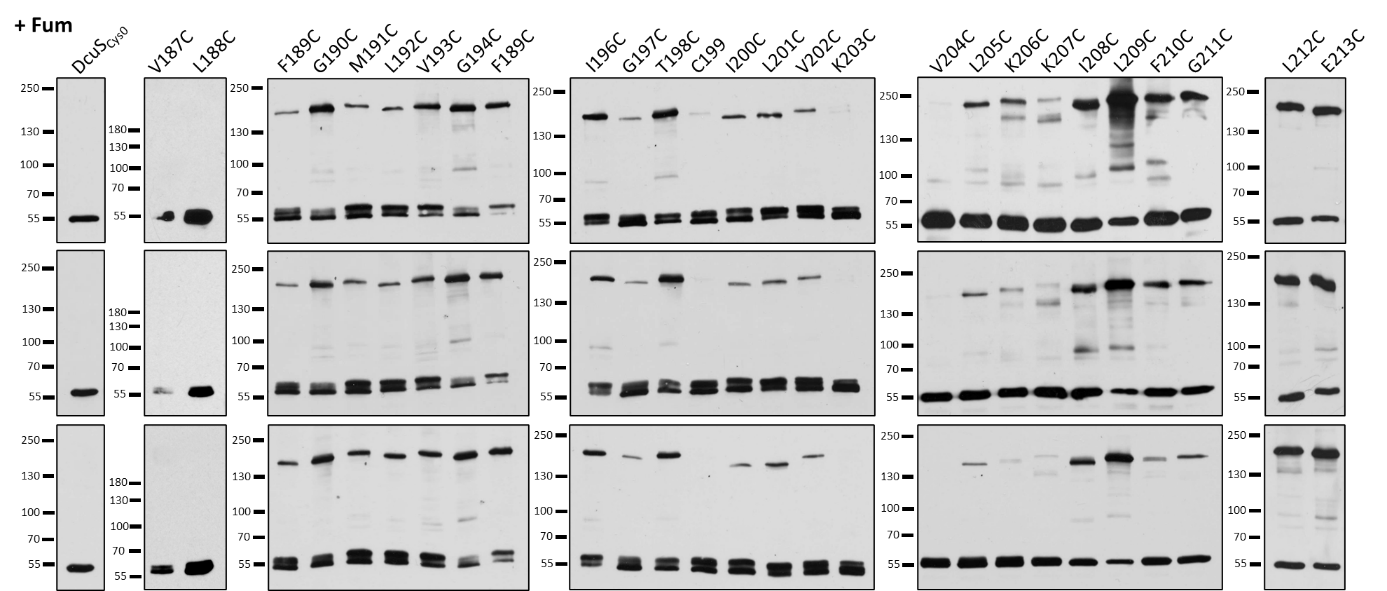


**Figure S8. Detection of DcuS after cross-linking of bacteria with Cu^2+^ phenanthroline in the presence of fumarate (SDS-PAGE and Western blot with anti-DcuS (PAS_P_).** Cell protein was separated in non reducing SDS-PAGE and DcuS and CL-products were detected by chemiluminescence after Western blotting as described (18). Scans of the X-ray films are shown. Each experiment was performed in biological triplicates that are arranged one below each other.


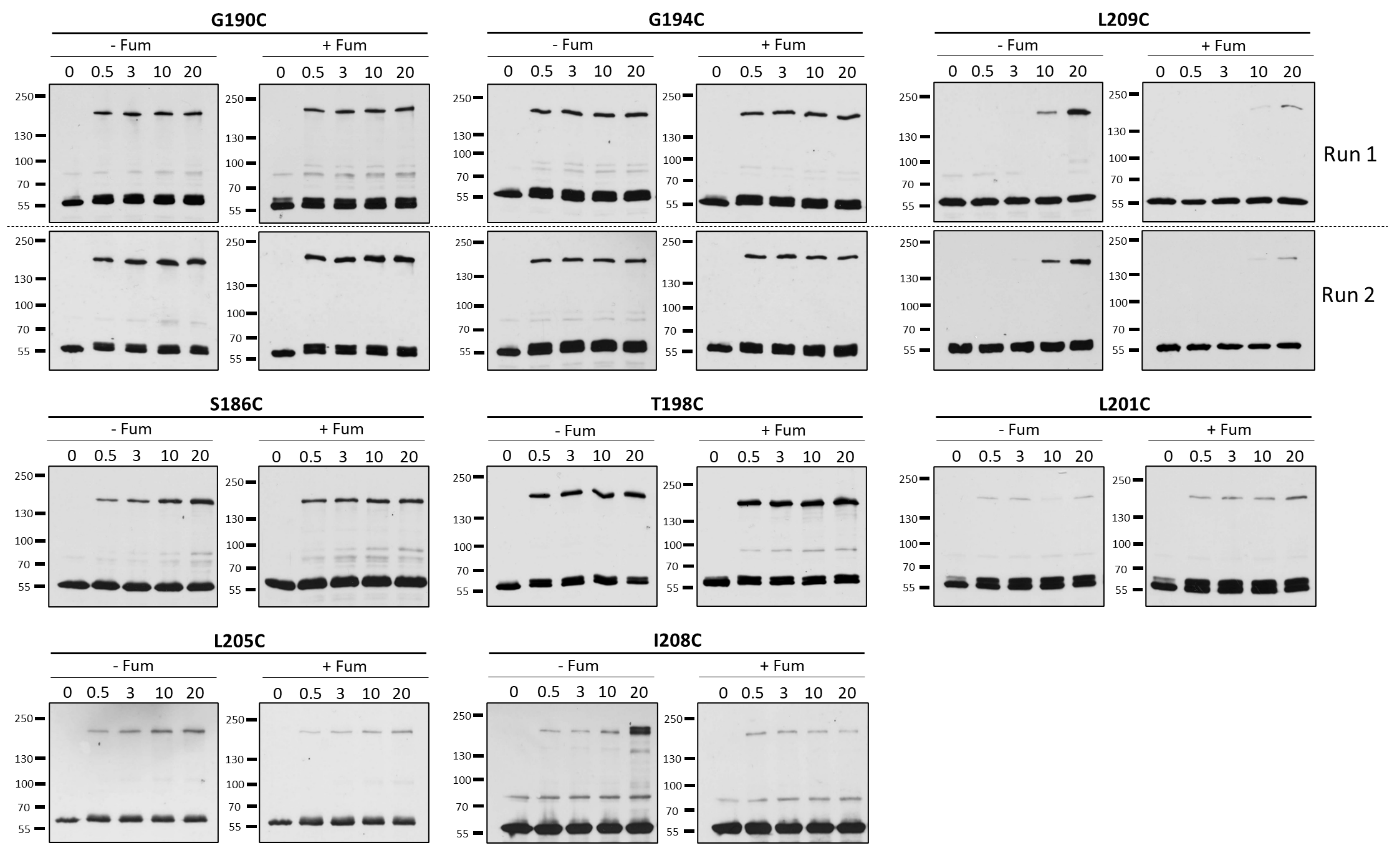


**Figure S9. Detection of DcuS after cross-linking of bacteria with Cu^2+^ phenanthroline (kinetics of cross-linking).** Cross-linking was performed for the reaction times shown above the scans (0 to 20 min) under the reaction conditions given in the Methods section, and in the absence (- Fum) or the presence (+ Fum) of fumarate. The samples were treated by SDS-PAGE and Western blotting as described for Figs. S8 and S9. Scans of the X-ray films are shown. For key residues G190C, G194C and L209C the was performed in repeat and are marked as Run 1/Run 2.

**Fig. S10. Schematic presentation of structural rearrangements in the domains of DcuS for transmembrane signaling after fumarate binding.** Dimeric DcuS (left side) and structural changes (right side) after fumarate binding are shown. The green arrows indicate the movement of α6 and TM2 after fumarate binding and binding pocket compaction of PAS_P_. The dashed arrows show the piston-type movement, the solid arrows the movement of the linker for signal conversion. Details are given in the main text

**Table S1.** *E. coli* strains and plasmids used in this study.

| **Strain or Plasmid** | **Genotype** | **Reference or source** |
| --- | --- | --- |
| *Eschrichia coli* K12 |  |  |
| C43(DE3) | Strain for overexpression of membrane proteins  carrying a chromosomal T7 polymerase | (45) |
| IMW260 | MC4100 λ[Φ(*dcuB’-‘lacZ*)*hyb, bla*^+^] *dcuS::cam^r^* | (22) |
| SU101 | *lexA71::Tn5, (Def)sulA211, Δ(lacIPOZYA)169/F’lacIqlacZΔM15::Tn9* | (53) |
| JM109 | *recA1, supE44, endA1, hsdR17, gyrA96, relA1, thi-1, Δ*(*lac-proAB*), [*F′, traD36, proAB, lacI^q^ZΔM15*] | (46) |
| BTH101 | F^-^, *cya-99, araD139, galE15, galK16, rpsL1 (Str^r^), hsdR2, mcrA1, mcrB1* | (49) |
| XL1-blue | *recA1, endA1, gyrA96, thi-1, hsdR17, supE44, relA1, lac, [F´ proAB lacIqZ∆M15 Tn10 (Tet^r^)]* | Stratagene |
| NT326 | *araDI39, ΔlacUl69, rpsL, thi, ΔmalE444, recA1* | (47) |
|  |  |  |
| Plasmids |  |  |
| pET28A | Expression vector, pBR ori, T7 Promoter, His_6_-tag (Kan^r^) | Novagene |
|  |  |  |
| Plasmids for *dcub-lacZ* assay | | |
| pMW181 | pET28a with *dcuS* (2.2 kb *Xba*I/*Hind*III fragment) (Kan^r^) | (28) |
| pMW778 | pMW181 but DcuS-L201A (Kan^r^) | This study |
| pMW809 | pMW181 but DcuS-S186C+G190A (Kan^r^) | This study |
| pMW813 | pMW181 but DcuS-G190C+G194A (Kan^r^) | This study |
| pMW819 | pMW181 but DcuS-G190A (Kan^r^) | This study |
| pMW827 | pMW181 but DcuS-S186C (Kan^r^) | This study |
| pMW841 | pMW181 but DcuS-G194A (Kan^r^) | This study |
| pMW852 | pMW181 but DcuS-W185A+F189A (Kan^r^) | This study |
| pMW852 | pMW181 but DcuS-G194A+T198A (Kan^r^) | This study |
| pMW1204 | pMW181 but DcuS-L188A (Kan^r^) | This study |
| pMW1205 | pMW181 but DcuS-L192A (Kan^r^) | This study |
| pMW1206 | pMW181 but DcuS-V193A (Kan^r^) | This study |
| pMW1207 | pMW181 but DcuS-L195A (Kan^r^) | This study |
| pMW1426 | pMW181 but DcuS-S186C+G194A (Kan^r^) | This study |
| pMW2808 | pMW181 but DcuS-K206A (Kan^r^) | This study |
| pMW2811 | pMW181 but DcuS-K207A (Kan^r^) | This study |
| pMW2814 | pMW181 but DcuS-L209A (Kan^r^) | This study |
| pMW2817 | pMW181 but DcuS-G211A (Kan^r^) | This study |
| pMW3045 | pMW181 but DcuS-V202A (Kan^r^) | This study |
| pMW3046 | pMW181 but DcuS-V204A (Kan^r^) | This study |
| pMW3047 | pMW181 but DcuS-I208A (Kan^r^) | This study |
| pMW3048 | pMW181 but DcuS-F210A (Kan^r^) | This study |
| pMW3059 | pMW181 but DcuS-C199A (Kan^r^) | This study |
| pMW3060 | pMW181 but DcuS-I200A (Kan^r^) | This study |
| pMW3061 | pMW181 but DcuS-K203A+K207A (Kan^r^) | This study |
| pMW3062 | pMW181 but DcuS-L205A+L209A (Kan^r^) | This study |
| pMW3063 | pMW181 but DcuS-K206A+L209A (Kan^r^) | This study |
|  |  |  |
| Plasmids for GALLEX TM-interaction assays | | |
| pBLM 100 | pBR322 derivative, expression plasmid for GALLEX chimeric proteins with N-terminal LexA and C-terminal MBP-fusion (Amp^r^) | (51) |
| pBLM GpA | LexA-TM_GpA[17]_-MBP expression plasmid, pBLM100 derivative (Amp^r^) | (51) |
| pBLM GpA-G83I | pBLM GpA derivative but G83I | (51) |
| pMW1960 | LexA-TM2_DcuS[20]_-MBP expression plasmid, pBLM100 derivative (Amp^r^) | This study |
| pMW1961 | LexA-TM2_DcuS[17]_-MBP expression plasmid, pBLM100 derivative (Amp^r^) | This study |
| pMW1963 | LexA-TM2_DcuS[16]_-MBP expression plasmid, pBLM100 derivative (Amp^r^) | This study |
| pMW1964 | pMW1963 but DcuS-G190A+G194A (Amp^r^) | This study |
| pMW1965 | pMW1963 but DcuS-S186C+G190A (Amp^r^) | This study |
|  |  |  |
| Plasmids for BACTH assays | | |
| pKT25-zip | T25-Zip expression plasmid, pUT18C derivative, (Amp^r^) | (48) |
| pMW426 | T25-DcuS expression plasmid, pKT25 derivative | (68) |
| pMW429 | T18-DcuS expression plasmid, pUT18C derivative | (68) |
| pMW1209 | pMW426 but DcuS-S186C+G190A (Kan^r^) | This study |
| pMW1210 | pMW429 but DcuS-S186C+G190A (Amp^r^) | This study |
| pMW1211 | pMW426 but DcuS-G190A+G194A (Kan^r^) | This study |
| pMW1212 | pMW429 but DcuS- G190A+G194A (Amp^r^) | This study |
| pMW1711 | pMW426 but DcuS-S186P (Kan^r^) | This study |
| pMW1712 | pMW429 but DcuS-S186P (Amp^r^) | This study |
| pMW1748 | pMW426 but DcuS-S186C (Kan^r^) | This study |
| pMW1750 | pMW426 but DcuS-G190A (Kan^r^) | This study |
| pMW1751 | pMW426 but DcuS-G194A (Amp^r^) | This study |
| pMW1752 | pMW429 but DcuS-S186C (Amp^r^) | This study |
| pMW1754 | pMW429 but DcuS-G190A (Amp^r^) | This study |
| pMW1755 | pMW429 but DcuS-G194A (Amp^r^) | This study |
|  |  |  |
| Plasmids for Cys cross-linking and *dcub-lacZ* assay | | |
| pMW151 | His_6_-DcuS expression plasmid, pET28a derivative (Kan^r^) | (35) |
| pMW325 | pMW151 but DcuS-C471S (Kan^r^) | (27) |
| pMW336 | pMW151 but DcuS-C199S-C471S (=DcuS_Cys0_) (Kan^r^) | (27) |
| pMW1588 | pMW336 but DcuS-D178C (Kan^r^) | (18) |
| pMW1589 | pMW336 but DcuS-S179C (Kan^r^) | (18) |
| pMW1590 | pMW336 but DcuS-R180C (Kan^r^) | (18) |
| pMW1591 | pMW336 but DcuS-R181C (Kan^r^) | (18) |
| pMW1592 | pMW336 but DcuS-S182C (Kan^r^) | (18) |
| pMW1593 | pMW336 but DcuS-I183C (Kan^r^) | (18) |
| pMW1594 | pMW336 but DcuS-I184C (Kan^r^) | (18) |
| pMW1595 | pMW336 but DcuS-W185C (Kan^r^) | (18) |
| pMW1596 | pMW336 but DcuS-S186C (Kan^r^) | (18) |
| pMW1597 | pMW336 but DcuS-V187C (Kan^r^) | (18) |
| pMW1598 | pMW336 but DcuS-L188C (Kan^r^) | (18) |
| pMW1840 | pMW336 but DcuS-I196C (Kan^r^) | (18) |
| pMW1841 | pMW336 but DcuS-G197C (Kan^r^) | (18) |
| pMW1842 | pMW336 but DcuS-T198C (Kan^r^) | (18) |
| pMW1843 | pMW336 but DcuS-I200C (Kan^r^) | (18) |
| pMW1844 | pMW336 but DcuS-L201C (Kan^r^) | (18) |
| pMW1845 | pMW336 but DcuS-V202C (Kan^r^) | (18) |
| pMW1846 | pMW336 but DcuS-K203C (Kan^r^) | (18) |
| pMW1847 | pMW336 but DcuS-V204C (Kan^r^) | (18) |
| pMW1848 | pMW336 but DcuS-L205C (Kan^r^) | (18) |
| pMW1849 | pMW336 but DcuS-K206C (Kan^r^) | (18) |
| pMW1850 | pMW336 but DcuS-K207C (Kan^r^) | (18) |
| pMW1851 | pMW336 but DcuS-I208C (Kan^r^) | (18) |
| pMW1852 | pMW336 but DcuS-L209C (Kan^r^) | (18) |
| pMW1853 | pMW336 but DcuS-F210C (Kan^r^) | (18) |
| pMW1854 | pMW336 but DcuS-G211C (Kan^r^) | (18) |
| pMW1855 | pMW336 but DcuS-L212C (Kan^r^) | (18) |
| pMW1856 | pMW336 but DcuS-E213C (Kan^r^) | (18) |
| pMW2132 | pMW336 but DcuS-V172C (Kan^r^) | (18) |
| pMW2133 | pMW336 but DcuS-T173C (Kan^r^) | (18) |
| pMW2134 | pMW336 but DcuS-Q174C (Kan^r^) | (18) |
| pMW2135 | pMW336 but DcuS-Q175C (Kan^r^) | (18) |
| pMW2136 | pMW336 but DcuS-I176C (Kan^r^) | (18) |
| pMW2137 | pMW336 but DcuS-N177C (Kan^r^) | (18) |
| pMW2521 | pMW336 but DcuS-F189C (Kan^r^) | This study |
| pMW2522 | pMW336 but DcuS-G190C (Kan^r^) | This study |
| pMW2523 | pMW336 but DcuS-M191C (Kan^r^) | This study |
| pMW2524 | pMW336 but DcuS-L192C (Kan^r^) | This study |
| pMW2525 | pMW336 but DcuS-V193C (Kan^r^) | This study |
| pMW2526 | pMW336 but DcuS-G194C (Kan^r^) | This study |
| pMW2527 | pMW336 but DcuS-L195C (Kan^r^) | This study |
|  |  |  |

| **Primer** | **Sequence 5’ - 3’** |
| --- | --- |
| TM1for(17)1 | GTA CCA CAG AGC TCC TTA ATG G |
| GALLEX-TM1v-17-rev | GAG AAC TAG TAA TCA GAT GCA CCA CCA AT |
| TM1for(16) | GTA CCA CGA GCT CGA TCT TAA TG |
| TM1rev(16) | GAG AAG TAA ACT AGT ATG CAC C |
| G-TM2_compl-for | GTC GCT GGA GCT CCA TTA TCT G |
| G-TM2_compl-rev | TCA GTA CTA GTA ACC AGA ATG CAG |
| TM2for(17)2 | TCG CTG GAG CTC TAT CTG GTC |
| TM2rev(17)2 | GTA CCT TAA CTA GTA ATG CAG GTG |
| TM2_17_rev | AAC CAG ACT AGT GGT GCC AAT C |

**Table S2.** Primer for construction of the TM2 GALLEX fusions.
